# Visible Light-Driven Photocatalysis of Al-Doped SrTiO_3_: Experimental and DFT Study

**DOI:** 10.3390/molecules29225326

**Published:** 2024-11-12

**Authors:** Ulzhan Abdikarimova, Madina Bissenova, Nikita Matsko, Aidos Issadykov, Igor Khromushin, Tatyana Aksenova, Karlygash Munasbayeva, Erasyl Slyamzhanov, Aigerim Serik

**Affiliations:** 1 Department of Materials Science, Nanotechnology and Engineering Physics, Satbayev University, Almaty 050032, Kazakhstan; ulzhanabdi01@gmail.com (U.A.);; 2 Institute of Nuclear Physics, Almaty 050032, Kazakhstanmunasbaeva@inp.kz (K.M.);; 3 Joint Institute for Nuclear Research, Dubna 141980, Russia; 4 Bes Saiman Group, Almaty 050057, Kazakhstan

**Keywords:** photocatalysis, SrTiO_3_, synthesis, photodegradation

## Abstract

Environmental problems associated with water pollution caused by organic dyes have raised serious concerns. In this context, photocatalytic processes have proven to be promising and environmentally friendly methods for water purification utilising abundant solar energy. In this study, a SrTiO_3_-based photocatalyst was modified by doping with Al ions and the deposition of dual co-catalysts (Rh/Cr_2_O_3_ and CoOOH) to enhance the photocatalytic decomposition efficiency of methylene blue (MB). Pure perovskite SrTiO_3_ was synthesised by chemical precipitation followed by calcination at 1100 °C. Al-doped SrTiO_3_ with deposited co-catalysts showed 3.2 times higher photocatalytic activity compared to unalloyed SrTiO_3_ with co-catalysts in MB decomposition under visible radiation. This study highlights the effectiveness of using dual co-catalysts and low-valence metal doping to enhance the efficiency of the photocatalytic decomposition of organic pollutants. The density functional theory analysis results show that the Al doping of SrTiO_3_ improves charge separation and increases the lifetime of photogenerated electrons and holes while maintaining the size of the forbidden band, which confirms its effectiveness for enhancing photocatalytic activity.

## 1. Introduction

In recent years, the pollution of aquatic ecosystems by organic substances including dyes, antibiotics, and pesticides has significantly worsened the environmental situation [1]. These pollutants not only disrupt aquatic ecosystems but also pose a threat to human health because they can accumulate in living organisms and cause serious diseases, including cancer [2]. Dyes used in textiles, cosmetics, plastics, and other industries are particularly prevalent, resulting in wastewater containing these substances [3,4]. Owing to their high stability, dyes such as methylene blue (MB) persist in aqueous environments and are difficult to degrade. MB is toxic and carcinogenic, thus posing a threat to human health and the environment. For example, Gahlot et al. [5] report cellular stress and apoptosis at elevated MB levels. Therefore, effective methods for its removal from water bodies must be developed [6]. Various methods are used to treat wastewater from dyes, such as electrocatalysis [7], coagulation–flocculation [8], ionic exchange [9], oxidation [10], biodegradation [11], and adsorption [12]. These technologies vary considerably in terms of their efficiency and environmental impact, and each has its own drawbacks. For example, adsorption, despite its efficiency, has several disadvantages, such as high investment, high operating costs, low efficiency, and the problems of recycling and reusability for actual application in wastewater treatment [13]. Similarly, ion exchange, though very effective in removing certain contaminants such as heavy metals or hardness ions, may possess a much lower effectiveness for other types of contaminants [14].

Over the past few decades, photocatalysis [15] has been extensively studied as an effective method for removing toxic substances and pathogens from water and air, producing hydrogen by splitting water, synthesising organic compounds for pharmaceuticals, treating cancer cells, and creating self-cleaning coatings. [16,17]. Photocatalysis is widely recognised as one of the most effective technologies for the efficient removal of toxic pollutants. The reaction, accelerated by a catalyst upon the absorption of light, is called photocatalysis. The photogeneration of e^−^/h^+^ pairs leads to the formation of hydroxyl and superoxide radicals, which interact with dye molecules and reduce pollutants [18]. To date, various options are known for composite structures used for the photocatalysis process, including CdZnS/TiO_2_ [19], GaN–ZnO [20], SrTiO_3_ [21], Y_2_Ti_2_O_5_S_2_ [22], PAN/SrTiO_3_ [23], BaTaO_2_N [24], TNT@SrTiO_3_ [25], and SrTaO_2_N [26]. However, most modern photocatalysts are limited by their poor efficiency, owing to excessive defects appearing in the crystal structure. Specifically, during the thermal treatment of SrTiO_3_, oxygen vacancies occur, provoking undesirable defects in the form of Ti^3+^, which act as recombination centres in the band structure. To solve this problem, previous studies on defect engineering [27] have shown that doping with low-valence metal cations at the B node is effective. As aforementioned, the replacement of Ti^3+^ by Ga^3+^ or Na^+^ to substitute Sr^2+^ can introduce oxygen vacancies into the perovskite structure, resulting in a decrease in the Ti^3+^ concentration associated with the deactivation of photocatalysis. In addition, the molten flux method, conducted at relatively high temperatures, allows for the efficient alloying of perovskites with low-valence metal cations while avoiding the formation of side impurities [28]. Most studies on the defect engineering of perovskite photocatalysts based on doped SrTiO_3_ have focused on the photocatalytic decomposition of water [29] and CO_2_ reduction [30]. However, studies on the photocatalytic decomposition of organic pollutants are limited, emphasising the need for further research in this area.

On the other hand, metal-doped SrTiO_3_, in its pure form, has shown limited efficiency in photocatalytic applications due to sluggish surface redox reactions and suboptimal charge separation. For instance, pure Al-doped SrTiO_3_ achieved only a 54.91% degradation of methylene blue over 5 h under UV light [31]. In another study, Al-doped SrTiO_3_ synthesised via the flux method achieved a 58% degradation of congo red under visible light within 90 min—a relatively extended interval considering the goals of photocatalytic applications [32]. These findings underscore the challenges associated with doped SrTiO_3_ in its pure form and highlight the need for further modifications to enhance activity [33]. To overcome these limitations, one effective approach is the deposition of cocatalysts and the construction of heterostructures. These strategies facilitate the efficient separation of photogenerated charges and introduce active reaction centres, thereby accelerating redox reactions on the photocatalyst surface. Similar studies have reported the improved photocatalytic degradation of organic dyes through such modifications, underscoring the potential of cocatalyst-decorated, doped SrTiO_3_ composites [32,34,35]. This work builds on these insights by examining the combined impact of Al doping and dual cocatalyst deposition on the photocatalytic degradation of methylene blue, aiming to bridge gaps in the current literature and demonstrate a more efficient degradation pathway.

**Figure 1:**
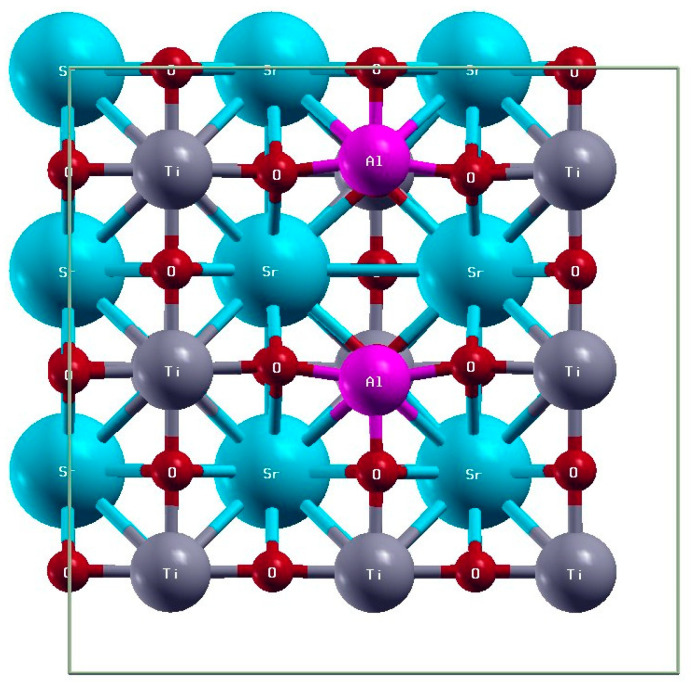
Elementary supercell of SrTiO_3_@Al.

In this study, Al-doped SrTiO_3_ photocatalyst SrTiO_3_ was successfully synthesised by the molten flux method followed by the photodeposition of dual co-catalysts (Rh/Cr_2_O_3_ and CoOOH). This photocatalyst exhibited high efficiency in the decomposition of MB under visible light. Highly crystalline SrTiO_3_ was prepared by a simple chemical precipitation method followed by calcination at 1100 °C. Various electron microscopy and structural analysis studies were carried out to evaluate the changes in the physicochemical properties of the photocatalysts before and after doping. The elementary supercell of SrTiO_3_@Al is shown in Figure 1. The analytical results indicate the promising application of dual co-catalysts and doping with low-valence metals to improve the efficiency of the photocatalytic decomposition of organic pollutants.

## 2. Results and Discussion

**Figure 2:**
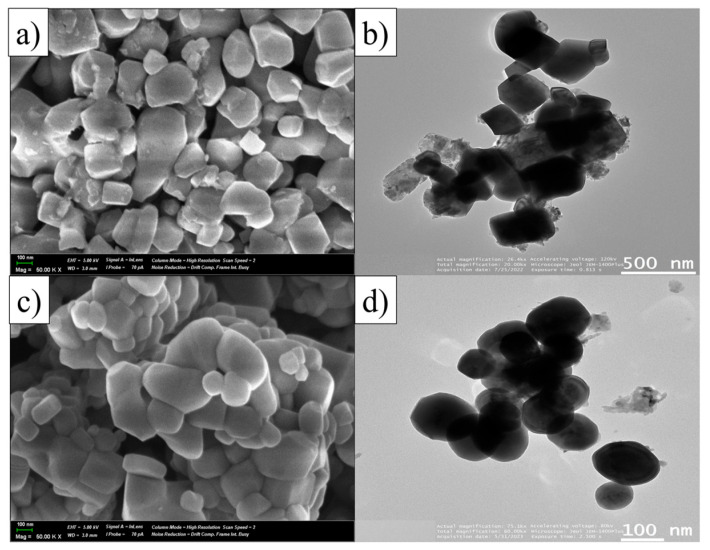
Morphology of samples: (**a**) SrTiO_3_ (1100 °C) obtained by SEM; (**b**) SrTiO_3_@Al obtained by TEM; (**c**) SrTiO_3_@Al obtained by SEM; and (**d**) SrTiO_3_@Al obtained by TEM.

Figure 2 presents the morphological images of SrTiO_3_ samples and doped SrTiO_3_@Al particles synthesised by the molten salt method at 1100 °C for 10 h, obtained using scanning electron microscopy (SEM) and transmission electron microscopy (TEM). The SrTiO_3_ samples shown in Figure 2a,b have well-defined block-like structures, with sizes ranging from 150 to 300 nm. The doping of SrTiO_3_@Al (Figure 2c,d) results in the particles acquiring more rounded faces and a homogeneous distribution, as seen in the TEM images. These particles exhibit a smooth, cubic morphology with sizes approximately between 100 and 150 nm. The cubic structure of SrTiO_3_@Al with truncated faces is formed because of the selective adsorption of Cl^−^ on the {111} faces when SrCl_2_ is used as flux. This adsorption lowers the surface energy of the facets, which in turn contributes to the expansion and reduction in the facet size {100} [36]. Further, EDX mapping confirmed the presence of Ti, O, Sr, and Al without any other impurities in the composition (Supplementary Figures S1 and S2).

As photocatalytic reactions occur on the catalyst surface, the presence of anisotropic crystalline faces with different surface energies significantly affects the photocatalytic activity. The anisotropic structure of cubic SrTiO_3_@Al improves the separation and transfer of photogenerated electrons and holes on the catalyst surface, which in turn promotes the formation of superoxide (•O_2_^−^) and hydroxyl (•OH) radicals in the reaction medium [37].

**Figure 3:**
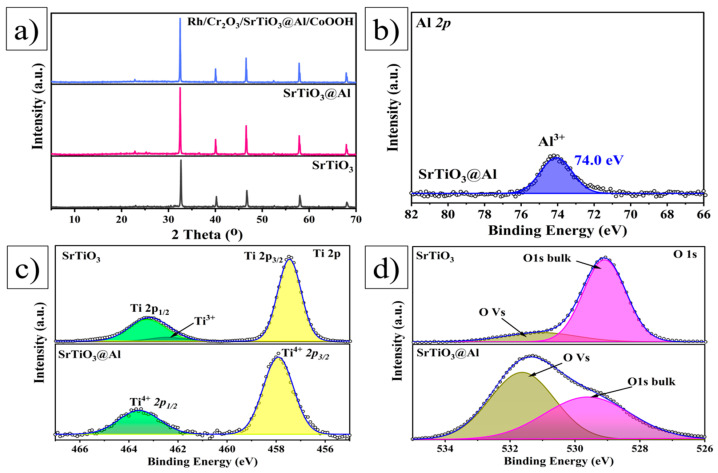
X-ray radiographs, synthesised photocatalysts of (**a**) SrTiO_3_, SrTiO_3_@Al, and Rh/Cr_2_O_3_/SrTiO_3_@Al/CoOOH; and XPS spectra of (**b**) Al 2p, (**c**) Ti 2 p, and (**d**) O 1s of SrTiO_3_@Al sample.

Figure 3a shows the X-ray diffraction (XRD) spectra of the SrTiO_3_, SrTiO_3_@Al, and Rh/Cr_2_O_3_/SrTiO_3_@Al/CoOOH samples. The spectrum of SrTiO_3_@Al shows the standard perovskite structure of the cubic SrTiO_3_ phase (JCPDS #35-0734) [38,39,40], which confirms the successful synthesis of the material. The comparison of the spectra of doped and undoped SrTiO_3_ shows a significant increase in the peak intensity of SrTiO_3_@Al, indicating improved crystallinity and increased crystal size because of Al doping performed using the molten salt method. In addition, the presence of aluminium and Rh/Cr_2_O_3_/CoOOH does not affect the crystal structure of SrTiO_3_, as their characteristic peaks are not observed in the spectrum. This may be due to their low content, high dispersibility, and low crystallinity [30,37]. X-ray photoelectron spectroscopy (XPS) is used to study the chemical composition and structure of the flux-doped SrTiO_3_@Al surface photocatalyst. Figure 3b–d present spectra showing the changes in chemical composition and elemental states in the Al-doped sample compared to pure SrTiO_3_. The Al 2p spectrum presented in Figure 3b shows a peak in the binding energy range of 72.1–76.4 eV, indicating the presence of Al^3+^. This confirms the incorporation of aluminium into the SrTiO_3_ lattice in the oxidised state through bonding with oxygen. Similar studies [41] indicate that high-temperature treatment causes oxygen vacancies in the SrTiO_3_ structure, leading to the formation of Ti^3+^ ions, as also shown in Figure 3c. In the undoped SrTiO_3_ sample synthesised at 1100 °C, Ti 2p peaks are observed corresponding to Ti^3+^ ions, which can serve as recombination centres [42]. In contrast, in the doped SrTiO_3_@Al sample, Ti^3+^ ions are completely absent, and the spectrum shows only Ti^4+^ peaks with binding energies of 457.9 and 463.6 eV. This indicates the stabilisation of titanium in the oxidised Ti^4+^ state. Figure 3d shows the O 1s level spectra, where two Gaussian peaks are revealed: one at 528.4 eV, which is associated with oxygen in the crystal lattice, and the other at 530.0 eV, corresponding to oxygen defects. Doping with Al significantly alters the ratio of the lattice and defect oxygen, increasing the defect oxygen content from 15.9% to 54.2% [21]. Thus, the results of XPS and XRD analysis confirm the successful synthesis of SrTiO_3_@Al. Al doping effectively suppresses the formation of Ti^3+^ ions and promotes the oxygen vacancies in an optimal amount. These findings agree with previous studies [43,44], which highlight the improved photocatalytic performance of SrTiO_3_ upon doping with aluminium.

### 2.1. Photocatalytic Performance

**Figure 4:**
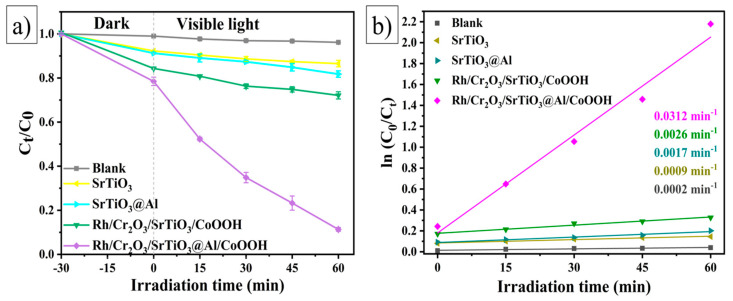
(**a**) Photocatalytic decomposition activity and (**b**) plots of ln$\left(\frac{C_{t}}{C_{0}}\right)$ versus time.

To evaluate the photocatalytic activity of the synthesised catalysts, experiments were performed on the photocatalytic degradation of the cationic dye MB in aqueous solution. In all the experiments, the samples were irradiated under visible light (λ ≥ 400 nm) for 60 min. The results illustrating the photocatalytic activity of the samples as a function of the irradiation time are presented in Figure 4a. The MB dye removal rates (initial concentration of 10 mg/L, 50 mL) were 13.5%, 18.2%, 27.9%, and 88.7% for the SrTiO_3_, SrTiO_3_@Al, Rh/Cr_2_O_3_/SrTiO_3_/CoOOH, and Rh/Cr_2_O_3_/SrTiO_3_@Al/CoOOH photocatalysts, respectively. The differences in photodegradation efficiency between SrTiO_3_ and SrTiO_3_@Al indicate the influence of the crystallite size effect related to Al doping, which was also discussed in the context of the sample morphology. An increase in the particle size can promote the recombination of photogenerated charges, which negatively affects the photocatalytic efficiency [45]. The highest photocatalytic activity was recorded for the Rh/Cr_2_O_3_/SrTiO_3_@Al/CoOOH sample, the efficiency of which exceeds that of SrTiO_3_, SrTiO_3_@Al, and Rh/Cr_2_O_3_/SrTiO_3_/CoOOH by a factor of 6.6, 4.9, and 3.2, respectively. This was attributed to the efficient charge separation and charge transfer provided by the dual separately photo-deposited co-catalysts in the catalyst/photocatalyst system.

According to a previous study [42], Al doping suppresses Ti^3+^ recombination centres, which promotes the spatial separation of photogenerated electrons and holes and their transfer to different active centres to participate in photocatalytic pollutant decomposition reactions [42].

The study of the kinetic characteristics plays an important role in understanding the mechanism of the photocatalytic decomposition of dyes. The pseudo-first-order model described in Equation (1) was used to estimate the rate of photocatalytic MB removal.

(1) $$-\ln(\frac{C_{t}}{C_{0}})=k_{p}t,$$

where C_0_ and C_t_ (mg/L) denote the initial and current dye concentrations at time t, and k_p_ is the reaction rate constant. Figure 4b shows linear plots of the dependence of $\left(\frac{C_{t}}{C_{0}}\right)$ on reaction time. The regression coefficient (R^2^) shows good correlation with the pseudo-first-order kinetics for different photocatalysts, as follows: R^2^ = 0.995 for SrTiO_3_, R^2^ = 0.972 for SrTiO_3_@Al, R^2^ = 0.980 for Rh/Cr_2_O_3_/SrTiO_3_/CoOOH, and R^2^ = 0.982 for Rh/Cr_2_O_3_/SrTiO_3_@Al/CoOOH. The calculations showed that the values of the constant k_p_ for SrTiO_3_, SrTiO_3_@Al, Rh/Cr_2_O_3_/SrTiO_3_/CoOOH, and Rh/Cr_2_O_3_/SrTiO_3_@Al/CoOOH are 0.0009, 0.0017, 0.0026, and 0.0312 min^−1^, respectively. The obtained data confirm that the Rh/Cr_2_O_3_/SrTiO_3_@Al/CoOOH sample is the most efficient photocatalyst for MB decomposition, which is consistent with the results of photodegradation studies.

### 2.2. Degradation Mechanism

Similar studies using radical scavengers such as isopropanol, benzoquinone, and EDTA to investigate the photocatalytic mechanism of SrTiO_3_ with cocatalysts have shown that superoxide (•O_2_^−^) and hydroxyl (•OH) radicals play a key role in the degradation of methylene blue [46,47].

The mechanism of action for the photocatalyst Rh/Cr_2_O_3_/SrTiO_3_@Al/CoOOH can be described as follows: upon the absorption of solar photons by SrTiO_3_@Al particles, electrons (e^−^) and holes (h^+^) are generated. The electrons are directed towards the reduction cocatalyst Rh/Cr_2_O_3_, while the holes migrate to the oxidation cocatalyst CoOOH. The strategic placement of these cocatalysts on different facets of SrTiO_3_@Al facilitates the effective separation of electron–hole pairs, thereby preventing their recombination and enabling redox reactions [48]. Superoxide radicals (•O_2_^−^) are formed as a result of the reaction between electrons and adsorbed oxygen, while hydroxyl radicals (•OH) are generated through the interaction of holes with water or hydroxide ions. Additionally, methylene blue absorbs photons, initiating the formation of reactive species. The interaction of these radicals with dye molecules leads to the production of CO_2_ and H_2_O, delineating the key stages of the photocatalytic reaction [21].

(2) $$Rh/{Cr}_{2}O_{3}/{SrTiO}_{3}@Al/CoOOH+h\nu \to e^{-}+h^{+}$$

(3) $${e^{-}+O}_{2}\to •O_{2}^{-}$$

(4) $${h^{+}+H}_{2}O\to •OH$$

(5) $$•OH+MB\to intermediate\;products\to CO_{2}+H_{2}O$$

(6) $$•O_{2}^{-}+MB\to intermediate\;products\to CO_{2}+H_{2}O$$

### 2.3. Density Functional Theory (DFT) Calculation

**Figure 5:**
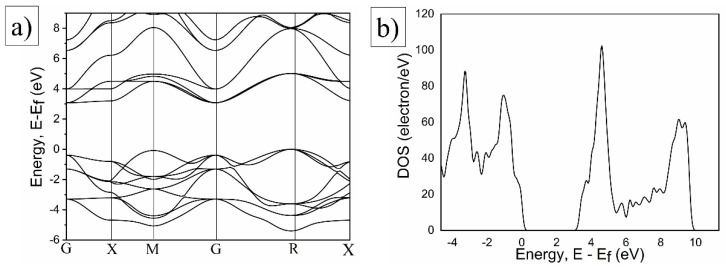
(**a**) DFT + U electronic band structure of bare SrTiO_3_; and (**b**) density of states of Al-doped SrTiO_3_.

To analyse the electronic structure of SrTiO_3_ and Al-doped SrTiO_3_, particularly in terms of band structure and density of states, DFT simulations were conducted. These results align with those of previous studies [49,50], indicating a calculated band gap of 1.99 eV using generalised gradient approximation (GGA), which is lower than the experimental value because of the well-known underestimation of on-site Coulomb interactions for d- and f-electrons. By incorporating the Hubbard U parameter (U = 5, J = 4), this limitation was mitigated, yielding a bandgap of 3.2 eV. Figure 5a illustrates the calculated band structure of pure SrTiO_3_, showing an indirect band gap of 3.19 eV, consistent with our previous experimental findings [21,51]. The anisotropy of the Ti 3d bands in the conduction zone was evident, with a nearly dispersionless character in the GX direction, whereas the quasiparticle energy increased rapidly in other directions. Thus, the excited electrons in the conduction band were likely to occupy states in the GX direction, where the kinetic energy was minimised. This corresponded to the distribution of conduction electron momenta perpendicular to the crystal’s cubic faces, as observed experimentally.

Al doping in SrTiO_3_ primarily replaces Ti atoms owing to the similar ionic radii of Al (54 pm) and Ti (61 pm) within the SrTiO_3_ lattice. The substitution of Ti^4+^ with Al^3+^ induces p-type conductivity by introducing impurity levels associated with Al near the valence band edge [52,53,54,55]. Our DFT calculations show that while Al doping does not significantly alter the band gap size, it does extend the lifetimes of photogenerated electrons and holes by reducing Ti^3+^ recombination centres. This finding is corroborated by XPS analysis (Figure 3), which indicates a decrease in recombination sites, thereby enhancing charge separation efficiency. Experimentally, this enhanced charge separation is evident in the photocatalytic performance of SrTiO_3_, as the prolonged lifetime of charge carriers reduces recombination and increases the availability of reactive species.

Consequently, the DFT results provide direct theoretical confirmation of the observed experimental behaviour, illustrating how Al doping modifies the electronic structure to extend charge carrier lifetimes and enhance the separation of photogenerated electron–hole pairs. Specifically, in the photocatalytic degradation of methylene blue, the SrTiO_3_@Al composite with deposited co-catalysts demonstrated a 3.2-fold improvement in efficiency compared to undoped SrTiO_3_ with an equivalent co-catalyst ratio. This enhanced performance is attributed to the increased charge separation and reduced recombination facilitated by Al doping, which is critical to achieving the observed degradation efficiency. These findings substantiate the superior photocatalytic activity of Al-doped SrTiO_3_, linking the DFT predictions to the improved experimental outcomes.

## 3. Methodology

### 3.1. Materials

Titanium (IV) oxide, anatase (TiO_2_, particle sizes < 25 nm, 99.7%), strontium nitrate (Sr(NO_3_)_2_, ≥ 98%), nitric acid (HNO_3_, < 90%), strontium titanate (SrTiO_3_, particle sizes < 100 nm, 99%), aluminium oxide (Al_2_O_3_, particle sizes < 50 nm, 99.8%), rhodium (III) chloride hydride (RhCl_3_·6H_2_O, Rh 38–40%), cobalt (II) nitrate hexahydrate (Co(NO_3_)_3_·6H_2_O, ≥98%), and methylene blue (C_16_H_18_ClN_3_S·xH_2_O, dye content, ≥82%) were purchased from Sigma–Aldrich (St. Louis, MO, USA). Oxalic acid ((COOH)_2_·2H_2_O, >98%), strontium chloride hexahydrate (SrCl_2_·6H_2_O, 99.7%), and potassium chromate (K_2_CrO_4_, 99.5%) were purchased from Laborpharma (Almaty, Kazakhstan). All chemicals were used without pretreatment.

### 3.2. Synthesis of SrTiO_3_ Calcined at 1100 °C

SrTiO_3_ powder was synthesised using a simple chemical precipitation method followed by calcination at 1100 °C as described in Ref. [56]. Briefly, a 0.12 M solution of Sr(NO_3_)_2_ was mixed with TiO_2_ in distilled water at a Sr/Ti molar ratio of 1:1 and subjected to ultrasonic treatment for 30 min. A 0.4 M solution of clear oxalic acid (COOH)_2_-2H_2_O was used as a reducing agent. The two solutions were combined in a chemical cylinder with constant stirring using a magnetic stirrer. Then, a 10% aqueous ammonia solution was added to the solution to achieve a pH of 6–7. The resulting precipitate was washed and dried at 60 °C for 16 h. In the final step, the product was calcined in a muffle furnace at 1100 °C for one hour, resulting in a white powder of SrTiO_3_.

### 3.3. Synthesis of SrTiO_3_@Al

The alloying of the SrTiO_3_ powder with aluminium was performed using the fluxing method. To synthesise SrTiO_3_@Al, SrTiO_3_, Al_2_O_3_, and SrCl_2_ powders were mixed in a molar ratio of 1:0.02:10 in an agate mortar for half an hour until a uniform mass was obtained. The mixture was then placed in an aluminium oxide crucible and heat-treated at 1100 °C in a muffle furnace for 10 h. After cooling, hot distilled water was added to the crucible and the mixture was ultrasonicated to separate the samples from the crucible. The precipitate was then washed with hot distilled water for five centrifugation cycles to remove residual SrCl_2_. The final step was drying the powder at 60 °C for 16 h to ensure the high purity of the final product.

### 3.4. Photodeposition of Double Co-Catalysts

SrTiO_3_ and SrTiO_3_@Al samples were further modified with dual co-catalysts, Rh/Cr_2_O_3_ and CoOOH, using the photodeposition method [21]. Then, 0.1 g of SrTiO_3_@Al powder was added to distilled water (50 mL) and subjected to ultrasonication for 30 min. The resulting suspension was placed in a reactor for photochemical reaction and 50 μL of RhCl_3_·6H_2_O was added, followed by irradiation (10 min) under constant magnetic stirring. Then, 25 μL of K_2_CrO_4_ (2 mg (Cr)/mL) and 25 μL of Co(NO_3_)_3_ (2 mg(Co)/mL) were added and irradiated for another 10 min. The resulting samples were washed several times and dried for 16 h at 60 °C. The amounts of added aqueous solutions of the co-catalysts were calculated for mass concentrations of Rh, Cr, and Co of 0.1%, 0.05%, and 0.05%, respectively. This allowed us to obtain samples with the photodeposition of the dual co-catalysts Rh/Cr_2_O_3_/SrTiO_3_@Al/CoOOH and Rh/Cr_2_O_3_/SrTiO_3_/CoOOH.

### 3.5. Characteristics

Data on the phase compositions of the obtained samples and modified composites were obtained using XRD using a Drone-8 with angles of 5–70° and steps of 0.01°. The morphology and elemental composition were studied using SEM (Zeiss Crossbeam 540 isCarl Zeiss Microscopy GmbH, Oberkochen, Germany) at 5–20 kV and energy-dispersive X-ray spectroscopy (EDX; INCA X-Sight). TEM (JEM-2100 LaB6 HRTEM) at 80 kV was used to study the morphologies of the co-catalysts. XPS was performed on a Microtech Multilab 3000 VG instrument with Mg and Al as X-ray sources for valence state analysis, calibrated by the C1s peak at 284.8 eV. Ultraviolet reflectance spectra (UV–Vis DRS) were recorded on a Perkin Elmer Lambda 35 spectrophotometer in the range of 200–800 nm.

### 3.6. Photocatalytic Test

The photocatalytic decomposition of MB in aqueous solution was carried out at room temperature (25 °C) using a photochemical reactor (Shanghai Leewen Scientific Instrument Co., Ltd., Shanghai, China) with a high-pressure mercury lamp (10 W; λ_max_ = 664 nm) and a cutoff filter (λ ≈ 400 nm). The distance between the quartz flask (50 mL) and lamp was set to 10 cm. The duration of photocatalytic irradiation was 60 min for each sample, and fractions (1 mL each) were collected every 15 min and filtered through a PVDF syringe filter. The resulting fractions were examined using the UV–Vis method (I5 Hanon Advanced Technology Group Co., Ltd., Jinan, China). All photocatalytic diagnostics were performed twice, and the mean values were considered to account for deviations.

### 3.7. Computational Details

The open-source Quantum ESPRESSO package was utilised for DFT calculations to investigate the electronic structure of SrTiO_3_ [57]. Al-doped SrTiO_3_ was modelled using a supercell consisting of 3 × 3 × 3 elementary cubic cells with a lattice parameter of 3.94 Å, as shown in Figure 1. In an ideal crystal, this supercell contains 135 atoms. Structural optimisation was performed using the Perdew–Burke–Ernzerhof functional within the GGA, with a plane-wave cutoff energy of 40 Ry and a 3 × 3 × 3 uniform k-point grid in the first Brillouin zone. The relaxation continued until the atomic forces were reduced below 10^−4^ Ry/Å. The standard DFT method encounters challenges in modelling d- and f-electron materials owing to self-interaction errors in the localised states. Therefore, the GGA + U method (Dudarev formulation) [58] was applied for electronic structure calculations on SrTiO_3_ and Al-doped SrTiO_3_. Given the hybridisation of Ti^−^ 3d and O^−^ 2p states [49,59,60], on-site Coulomb corrections, with U values of 5.9 eV for Ti and 4.2 eV for O, were applied to ensure accuracy. The cutoff energy was set to 60 Ry and a 5 × 5 × 5 k-point grid was used for the calculations.

## 4. Conclusions

In this study, the photocatalytic activity of synthesised SrTiO_3_-based catalysts was evaluated for MB degradation under visible light. The Rh/Cr_2_O_3_/SrTiO_3_@Al/CoOOH sample showed the best results, with the dye removal efficiency reaching 88.7% within 60 min, which exceeds the activity of Rh/Cr_2_O_3_O_3_/SrTiO_3_/CoOOH by 3.2 times. These results indicated that Al doping reduces Ti^3+^ recombination centres and promotes the efficient separation and transfer of photogenerated charges. Kinetic studies confirmed the photocatalytic decomposition mechanism as pseudo-first-order, with a decomposition rate constant of 0.0312 min^−1^ for the Rh/Cr_2_O_3_/SrTiO_3_@Al/CoOOH sample, highlighting its outstanding efficiency. DFT analysis revealed that the Al doping of SrTiO_3_ improves charge separation and extends the lifetime of photogenerated electrons and holes while maintaining the bandgap size. However, to achieve substantial enhancement in photocatalytic activity, additional measures such as the use of dual co-catalysts are required. These co-catalysts create active centres and facilitate effective charge transfer, significantly boosting pollutant degradation efficiency.

## Data Availability

Data are contained within the article.

## Supplementary Materials

The following supporting information can be downloaded at: https://www.mdpi.com/article/10.3390/molecules29225326/s1, Figure S1: EDX mapping of SrTiO_3_@Al; Figure S2: TEM images.
