# Supplementary material for: Visible Light-Driven Photocatalysis of Al-Doped SrTiO3: Experimental and DFT Study
Source: Molecules. 2024 Nov 12;29(22):5326. doi: 10.3390/molecules29225326 (PMC11596584; doi:10.3390/molecules29225326)
Supplement: Supplementary file 1 [file molecules-29-05326-s001.zip › molecules-3290716-supplementary.pdf]

Visible Light-Driven Photocatalysis of Al-Doped $SrTiO_3$: Experimental and DFT Study

Ulzhan Abdikarimova [1,2], Madina Bissenova [1,2], Nikita Matsko [3], Aidos Issadykov [2,3], Igor Khromushin [2], Tatyana Aksenova [2], Karlygash Munasbayeva [2], Erasyl Slyamzhanov [2] and Aigerim Serik [1,2,4,*]

[1] Department of Materials Science, Nanotechnology and Engineering Physics, Satbayev University, Almaty 050032, Kazakhstan
[2] Institute of Nuclear Physics, Almaty 050032, Kazakhstan
[3] Joint Institute for Nuclear Research, Dubna 141980, Russia
[4] Bes Saiman Group, Almaty 050057, Kazakhstan
* Correspondence: aigerim.serik3508@gmail.com; Tel.: +7-7076050464

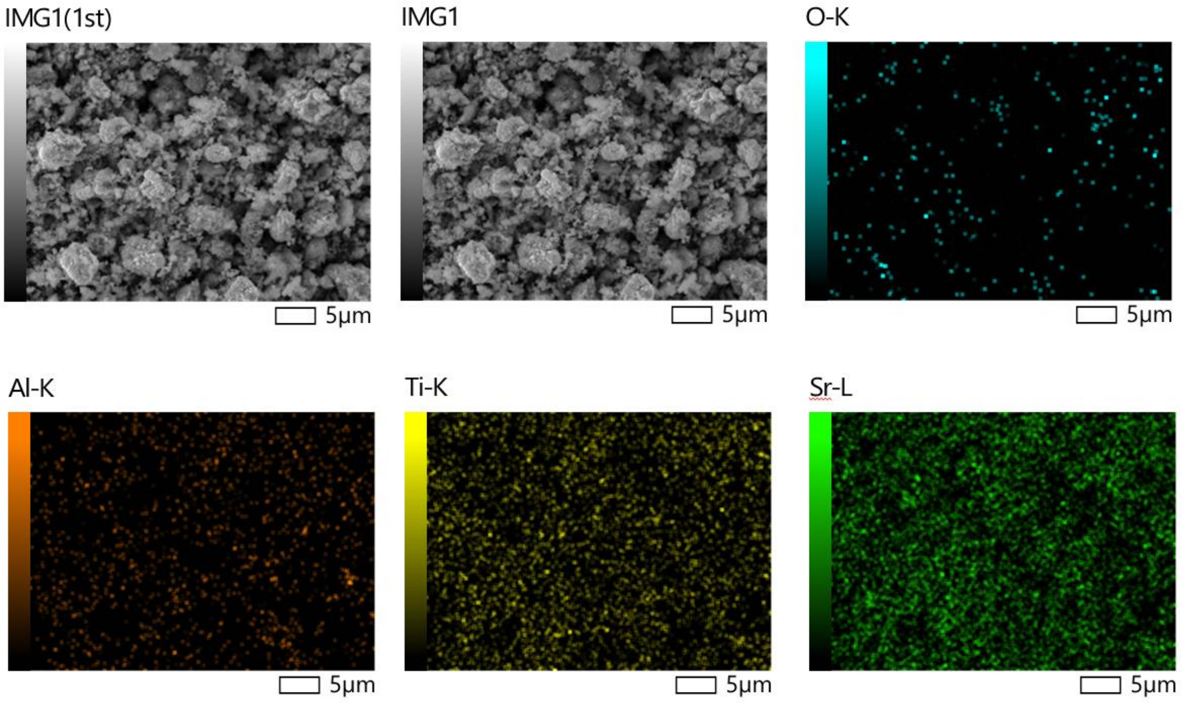

Figure S1. EDX mapping of $SrTiO_3$@Al

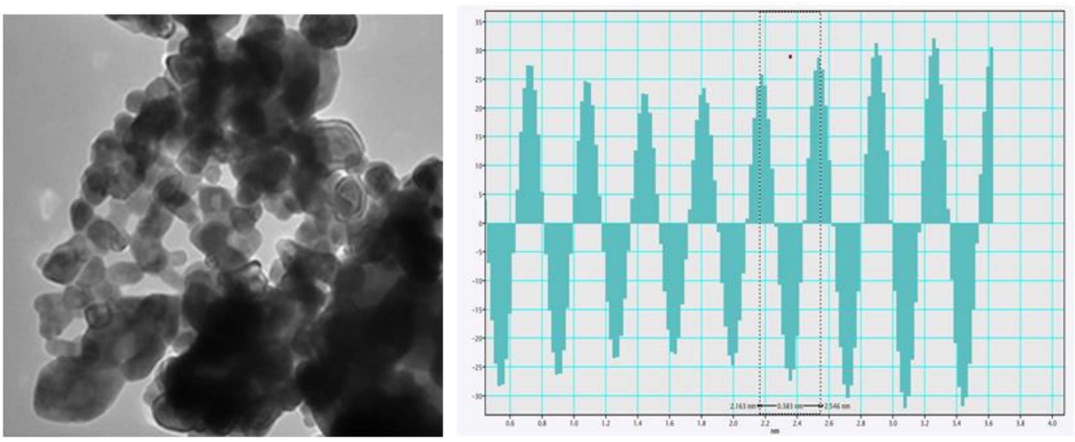

Figure S2. TEM images
